# A Squalene-Based Nanoemulsion for Therapeutic Delivery of Resiquimod

**DOI:** 10.3390/pharmaceutics13122060

**Published:** 2021-12-02

**Authors:** Zhongkun Zhang, Jimmy Chun-Tien Kuo, Chi Zhang, Yirui Huang, Zerui Zhou, Robert J. Lee

**Affiliations:** Division of Pharmaceutics and Pharmacology, College of Pharmacy, The Ohio State University, Columbus, OH 43210, USA; zhang.5763@osu.edu (Z.Z.); kuo.249@osu.edu (J.C.-T.K.); zhang.9395@osu.edu (C.Z.); huang.4650@osu.edu (Y.H.); zhou.3561@osu.edu (Z.Z.)

**Keywords:** nanoemulsions, TLR agonists, solid tumors, SD-101, resiquimod

## Abstract

Agonists for toll-like receptors (TLRs) have shown promising activities against cancer. In the present study, a squalene-based nanoemulsion (NE) was loaded with resiquimod, a TLR7/8 agonist for therapeutic delivery. R848 NE was developed and characterized for long-term stability. In vitro and in vivo antitumor immunity of R848 NE were also evaluated in combination with SD-101, a CpG-containing TLR9 agonist. In vitro studies demonstrated strong long-term stability and immune responses to R848 NE. When combined with SD-101, strong antitumor activity was observed in MC38 murine colon carcinoma model with over 80% tumor growth inhibition. The combination treatment showed a 4-fold increase in systemic TNFa production and a 2.6-fold increase in Cd8a expression in tumor tissues, suggesting strong cell-mediated immune responses against the tumor. The treatment not only demonstrated a strong antitumor immunity by TLR7/8 and TLR9 activations but also induced PD-L1 upregulation in tumors, suggesting a potential therapeutic synergy with immune checkpoint inhibitors.

## 1. Introduction

Toll-like receptors (TLRs) play critical roles in immune responses by recognizing pathogen-associated molecule patterns (PAMP) followed by inducing cytokine production and activating adaptive immunity. TLRs are expressed either on the plasma membrane (TLR1/2/4/5/6/10) or in endosomes (TLR3/7/8/9) in antigen-presenting cells (APCs) such as dendritic cells and macrophages. TLR activation leads to the MyD88/NF-κB pathway induction and naïve T cell repertoires activation in adaptive immune responses [1]. Studies have shown that endosomal TLR agonists worked effectively as adjuvants in cancer vaccines due to their strong immunostimulatory activities [2]. Endosomal TLR agonists have been shown to activate plasmacytoid dendritic cells (pDCs) and cytotoxic T lymphocytes (CTLs), enhancing T cell-mediated immunity. Three agents with TLR agonist activity have been approved by the FDA for cancer treatments including bacillus Calmette-Guerin (TLR2&4 agonists mixture), monophosphoryl lipid A (TLR2/4 agonists mixture), and imiquimod (TLR7 agonist) [2]. Overall, the clinical efficacy of TLR agonists has been mixed [3,4]. Intratumoral injection has been used in recent clinical trials of CpG TLR9 agonists. However, this mode of administration is difficult in clinical practice for most solid tumors.

Previous studies have demonstrated strong innate and adaptive immune system activation and promising antitumor efficacy using TLR7/8 and TLR9 combinations [4,5,6]. Synergistic cytokine release and antibody productions were observed using a Schistosoma japonicum DNA vaccine, containing a combination of TLR 7/8 and TLR9 agonists [5]. Another study has shown significant tumor suppression and synergistic IFN-γ secretion by TLR7/8/9 combination treatments [5,6,7,8]. However, these studies on duo-TLR activation lacked an efficient platform for the delivery of TLR agonists.

Resiquimod (R848) is a TLR7/8 agonist that has shown antitumor activity in murine tumor models [7,8,9,10]. However, free R848 is practically insoluble in water, which means that, to make it an injectable agent, a solubilizing vehicle is required. DMSO is an organic solvent that is unsuitable for injection. In the clinic, Cremophor EL is known to cause hypersensitivity reactions when injected [11]. Therefore, R848 requires the development of an injectable formulation for clinical practice. An R848-loaded PLA-based nanoparticle has been developed for cancer immunotherapy recently [12]. However, the in vivo antitumor activity of these nanoparticles has not been reported. Oil-in-water nanoemulsions (NE) are effective delivery systems for hydrophobic drugs [13,14,15,16]. NE consists of an oil core stabilized by surfactants, where the oil core could work as an efficient reservoir for poorly water-soluble drugs [17]. In addition, squalene-based NE has been shown as an efficient vaccine adjuvant by adaptive immunity activation [18]. Squalene-based NE vaccine adjuvants MF59 (Novartis AG, Basel, Switzerland) and AS03 (GlaxoSmithKline plc, London, United Kingdom) have been administered to more than 100 million people in more than 30 countries, in both seasonal and pandemic influenza vaccines. It is also noticed that the squalene core in NE, which was originally derived from shark liver oil, has been reported to potentiate both immune responses and antitumor efficacy [19].

**Figure 1:**
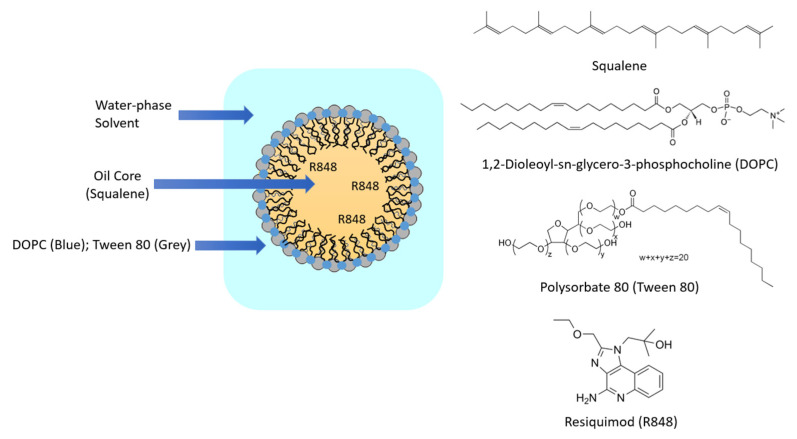
Structure of R848-loaded squalene emulsion and chemical components.

In this study, a squalene-based NE was synthesized using 1,2-dioleoyl-*sn*-glycero-3-phosphocholine (DOPC) and polysorbate 80 (Tween 80) as surfactants to deliver R848 (Figure 1). Here, the squalene core was utilized not only to potentiate immune response but also to deliver R848. DOPC and Tween 80 were utilized to provide particle stability and have already been incorporated in lipid-based nanoparticles in clinical trials [20]. R848 encapsulation efficiency in squalene-based NE received optimized drug-loading capacity by adjusting the lipid-to-drug ratio. The R848-loaded squalene-based NE showed long-term stability up to 1 month at 4 °C. R848 has been investigated for topical use in clinics, which limits its broad application against cancer [21]. The present study demonstrated a strong antitumor activity of R848-loaded squalene-based NE through systemic administration, which provides a novel administration route for R848 and might be applicable to other hydrophobic drugs. This new therapeutic strategy of R848 could greatly expand the indications of R848.

SD-101 is a class-C TLR9 agonist containing cytidine-phospho-guanosine (CpG) dinucleotides. It has been shown to potentiate both innate and adaptive immune responses [22]. Furthermore, SD-101 has been shown promising antitumor efficacy in combination with immune checkpoint inhibitors and radiation therapies in clinical trials [22,23,24]. Our study demonstrated the synergistic antitumor activity through adaptive immune stimulation by R848-loaded NE and SD-101. The combination treatment of R848-loaded NE and SD-101 also showed upregulation of the *Pdl1* mRNA level in a mouse model, suggesting a potential strategy of combination with anti-PD-L1 for cancer therapy.

## 2. Materials and Methods

### 2.1. Materials

Squalene was obtained from Sigma-Aldrich (St. Louis, MO, USA). 1,2-Dioleoyl-sn-glycero-3-phosphocholine (DOPC) was purchased from Avanti Polar Lipids (Birmingham, AL). Resiquimod (R848) was purchased from MedChem Express (Monmouth Junction, NJ, USA), and SD-101 was synthesized by Alpha DNA (Montreal, QC, Canada). TNFa, IL-6, and IL-12p70 mouse uncoated ELISA kits and high-capacity cDNA reverse transcription kit were purchased from Invitrogen (Waltham, MA, USA). SsoAdvanced^TM^ Universal SYBR^®^ Green Supermix was purchased from Bio-Rad Laboratories (Hercules, CA, USA). Real-time PCR pre-designed primers for murine Pdl1 (Cd274), Calreticulin, Hmgb1, and Actb were obtained from Sigma-Aldrich (St. Louis, MO, USA). Primers for murine Cd3e, CD4, CD8a, Foxp3, and Ifnγ were designed (Table S1) and synthesized by ThermoFisher Scientific (Waltham, MA). Polysorbate 80 (Tween 80) and all other chemicals and buffers otherwise stated were purchased from Fisher Scientific (Hampton, NH, USA).

### 2.2. R848-NE Formulation and Characterization

Squalene-based NE was prepared by rapid injection of an ethanol solution of the oil-lipid mixture into phosphate-buffered saline (PBS). Squalene, DOPC, and Tween 80 were prepared at a molar ratio of 1:1:1 in ethanol. R848 was then added to the lipid-ethanol solution, maintaining the lipid-to-R848 ratio at 10:1 (*w/w*). The final total lipid concentration of the nanoemulsion was 8 mg/mL, and the final R848 concentration was 0.8 mg/mL. R848-loaded NE (R848 NE) with lipid to R848 weight ratios of 20:1, 15:1, 10:1, 5:1, and 2:1 were prepared by increasing the amount of R848 added into the lipid mixture prior to NE preparation. Particle sizes were measured by dynamic light scattering (DLS) using a Nicomp Nano ZLS Z3000 (Entegris, Billerica, MA, USA). Empty nanoemulsion (empty NE) was generated using similar procedures without adding R848. Empty NE and R848 NE were stored at 4 °C before characterization.

Sepharose CL-4B size exclusion chromatography was performed to examine the encapsulation efficiency of R848 within the squalene nanoemulsions. R848 concentrations were determined by UV-Vis spectrometry at 320 nm using a NanoDrop 2000 spectrophotometer [25]. R848 loading efficiency was determined by Equation (1):

(1) $$Loading\;Efficiency\;\%=\frac{UV\;absorbance\;at\;320\;nm\;of\;fractions\;3–6\times volume}{UV\;absorbance\;at\;320\;nm\;of\;all\;fractions\times volume}\times 100$$

### 2.3. Cell Culture

RAW 264.7 murine macrophage and MC38 murine colorectal carcinoma cell lines were kind gifts from Dr. Peixuan Guo and Dr. Christopher Coss, respectively, at The Ohio State University College of Pharmacy. RAW 264.7 and MC38 were grown in DMEM supplemented with 10% FBS and 1 x antibiotic-antimycotic and maintained at 37 °C under a humidified atmosphere containing 5% CO_2_.

### 2.4. In Vitro Macrophage Stimulation Imaging

RAW 264.7 cells were seeded at a density of 1.5 × 10^5^ cells/well in 24-well plates 24 h before treatments. Cells were treated with empty NE, R848, SD-101, R848 NE, or R848 NE/SD-101 combination for 24 h. R848 was treated at 50 µM either as a single agent, in nanoemulsion, or in combination with SD-101. SD-101 was treated at 300 nM individually or in combination. The morphological changes of RAW 264.7 cells were visualized under 200× brightfield by a Nikon Eclipse Ti-S microscope (Nikon, Tokyo, Japan) after 24-h incubation.

### 2.5. In Vitro Cytokine Induction Evaluation by Enzyme-Linked Immunosorbent Assay (ELISA)

RAW 264.7 cells were seeded at a density of 3 x 10^5^ cells/well in 6-well plates 24 h before treatments. Cells were treated with empty NE, R848, SD-101, R848 NE, or R848 NE/SD-101 combination for 24 h. R848 was treated at 50 µM either individually, in nanoemulsion, or the combination. SD-101 was treated at 300 nM either individually or in combination. The supernatant was collected and stored at −80 °C followed by cytokine quantification by ELISA. Supernatants were 6-fold pre-diluted by PBS, and TNFa, IL-6, and IL-12p70 concentrations were measured by mouse uncoated ELISA kits per manufacturer’s protocol.

### 2.6. In Vivo Antitumor Efficacy

MC38 murine colorectal syngeneic model was generated by subcutaneously inoculating C57BL/6N mice (obtained from Charles River Laboratories) with 0.5 × 10^6^ cells per mouse on the right flack. Treatments were initiated once tumors reached approximately ~100 mm^3^. Mice (*n* = 5) were intraperitoneally treated with saline, 4 mg/kg R848 NE, 2 mg/kg SD-101, or R848 NE/SD-101 combination (4 mg/kg R848 NE and 2 mg/kg SD-101). All treatment solutions were prepared in PBS. Mice were dosed every 3 days for 4 doses. Tumor growth and body weight were monitored, and the tumor volumes were calculated according to Equation (2):

(2) $$Tumor\;Volume=\frac{Length\times Width^{2}}{2}$$

All animal studies were reviewed and approved by The Ohio State University Institutional Laboratory Animal Care and Use Committee (IACUC). All mice were euthanized on day 10, 6 h after the fourth dose to peak the serum cytokine concentrations. Whole blood was collected through cardiac puncture. Tumor and spleen tissues were harvested and weighed for comparison. Spleen weights were normalized to individual body weights for comparison between treatment groups. Tissues and sera were stored at −80 °C before in vivo cytokine and gene regulation studies. Tumor growth inhibition (%TGI) on day 10 was determined by Equation (3):

(3) $$\%TGI=\frac{1-\left(T_{10}/T_{0}\right)/\left(C_{10}/C_{0}\right)}{1-C_{0}/C_{10}}\times 100\%$$

where *T*_10_ stands for average tumor volume of treatment group at day 10, *T*_0_ stands for average tumor volume of treatment group at day 0, *C*_10_ stands for average tumor volume of the control group at day 10, and *C*_0_ stands for average tumor volume of the control group at day 0. %TGI > 50% was considered meaningful.

### 2.7. In Vivo Cytokine Measurement

Mouse sera were collected by placing whole blood at room temperature for 30 min followed by 2000× *g* centrifugation for 20 min. Samples were collected and stored at −80 °C before cytokine quantification. Murine TNFa, IL-6, and IL12p70 cytokine concentrations were determined by ELISA per the manufacturer’s protocol.

### 2.8. In Vivo Gene Regulation by Real-Time qPCR

Tumor and spleen tissues were homogenized in TRI reagent using probe sonication, and total RNA was extracted per the manufacturer’s protocol. cDNA was prepared using a High-Capacity cDNA Reverse Transcription Kit per manufacturer’s protocol. Real-time PCR was conducted on a QuantStudio 7 Flex Real-Time PCR System with target genes *Pdl1*, *Foxp3*, *Ifng*, *Cd3e*, and *Cd8a* in spleen tissue samples and *Pdl1*, *Calreticulin*, *Hmgb1*, *Cd3e*, *Cd4*, and *Cd8a* in tumor tissue samples. All genes were normalized to *Actb* as the housekeeping gene. The relative amount of RNA level was calculated and compared according to the 2^−^^ΔΔCt^ method [26,27].

### 2.9. Statistical Analysis

All studies were done in triplicate. Data are presented as means ± standard deviations unless otherwise indicated. Statistical analysis was conducted using Microsoft Excel. One-way ANOVA was used to determine variances in means between two or more treatment groups. Student’s *t* test was used as a post-hoc analysis to determine statistically significant differences between any two groups. A *p*-value of 0.05 was selected as the cutoff for statistical significance.

## 3. Results and Discussion

### 3.1. Particle Characterization

**Figure 2:**
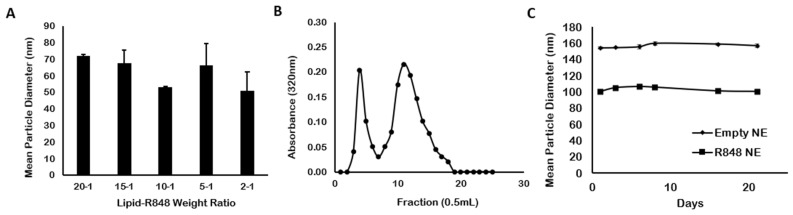
Particle characterizations of R848 NEs. (**A**) Particle sizes of R848 NEs with lipid-to-R848 weight ratio of 20:1, 15:1, 10:1, 5:1, and 2:1. (**B**) SEC chromatogram of R848 NE using a Sepharose CL-4B gel column. Absorbance at 320 nm was measured for the presence of R848. (**C**) Particle stability of empty NE and R848 NE stored at 4 °C, up to 3 weeks.

As a TLR7/8 agonist, R848 has demonstrated high antitumor activity compared with other imidazoquinoline analogs [28]. However, the tolerance induction and adverse effects limit its development as a candidate for clinical development. Polymer-based nanoparticles such as polylactic acid (PLA) or β-cyclodextrin have been studied as carriers of R848 to overcome these limitations [12,29,30]. Nonetheless, the disadvantages of polymeric nanoparticles include degradation for polymer materials and self-aggregation [29]. A squalene-based oil-in-water NE was prepared as a carrier for R848. R848 NE was approximately 50–100 nm in size (Figure 2A). There were no significant changes in particle sizes among lipid-to-R848 weight ratio from 20:1 to 2:1, indicating the addition of R848 not affecting the structural stability of the nanoemulsions. However, R848 precipitation due to insufficient oil phase was observed in R848 NE samples with 2:1 lipid-to-R848 weight ratio after storing overnight at 4 °C and with 5:1 lipid-to-R848 weight ratio after 1-week storage at 4 °C (data not shown). Therefore, R848 NE with a 10:1 lipid-to-R848 weight ratio was selected for further studies with maximized R848 loading amount. Size exclusion chromatography using a Sepharose CL-4B gel column (Figure 2B) showed 35.9 ± 0.53% of R848 within the NE-encapsulated fractions, which was higher than another liposomal formulation of R848 with only 7% encapsulation efficiency [30]. The result indicated that oil-in-water nanoemulsion worked better than liposomes in encapsulating the poor water-soluble agent. We further demonstrated that both empty NE and R848 NE exhibited high colloidal stability under storage at 4 °C over a period of 3 weeks (Figure 2C), with particle sizes of empty NE and R848 NE remained constant at 6-month measurement (Table S2). However, there was an unexpected particle size shrinkage between empty NE and R848 NE where R848 NE showed approximately 50 nm smaller than the empty NE in median particle diameter (R848 NE ~ 100 nm, empty NE ~ 150 nm). The reduction in particle sizes after R848 loading may result from the hydrophilic interactions between R848 and the aqueous phase, which decreased the hydrophobic interactions between emulsion particles and the aqueous phase. Nonetheless, the particle size of R848 NE with 50–100 nm is considered suitable for cellular uptake in antigen-presenting cells based on published studies [31,32].

### 3.2. In Vitro Macrophage Stimulation

**Figure 3:**
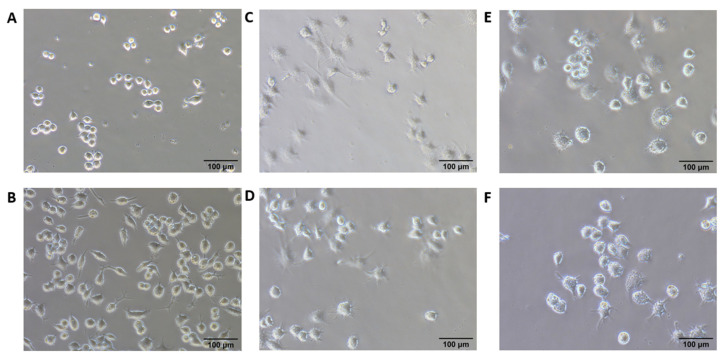
200X brightfield images of RAW 264.7 cells. RAW264.7 cells were treated with (**A**) complete medium only, (**B**) empty NE, (**C**) free R848, (**D**) SD-101, (**E**) R848 NE, and (**F**) R848 NE with SD-101 for 12 h. R848 was treated at 50 µM individually, in emulsion, or in combination. SD-101 was treated at 300 nM individually or in combination.

Previous research demonstrated that morphological changes of macrophages upon activation could be visualized under the microscope, which could further be utilized to study the factors modulating pro-inflammatory (M1) and anti-inflammatory (M2) activation [33,34]. TLR7, 8, and 9 are expressed majorly on pDCs and macrophages [28,35,36,37,38,39], which further polarizes naïve macrophages to M1 activation [40,41]. Classically polarized M1 macrophages produce pro-inflammatory and immunostimulatory cytokines which are crucial for tumor cell killing [1]. RAW 264.7 murine macrophage cells were utilized to examine the immune stimulation carried out by R848 NE treatment as well as addition with SD-101 treatment. Our result showed that untreated RAW 264.7 generally exhibited a round form, whereas empty NE-treated RAW 264.7 exhibited a round form but began forming filopodia (Figure 3A,B). R848-treated and SD-101-treated RAW 264.7 showed partially activated macrophages with partial expansion and lamellipodia formation (Figure 3C,D). Finally, RAW 264.7 treated with R848 NE and R848 NE/SD-101 combination showed fully activated macrophages with accelerated spreading and lamellipodia formation (Figure 3E,F).

**Figure 4:**
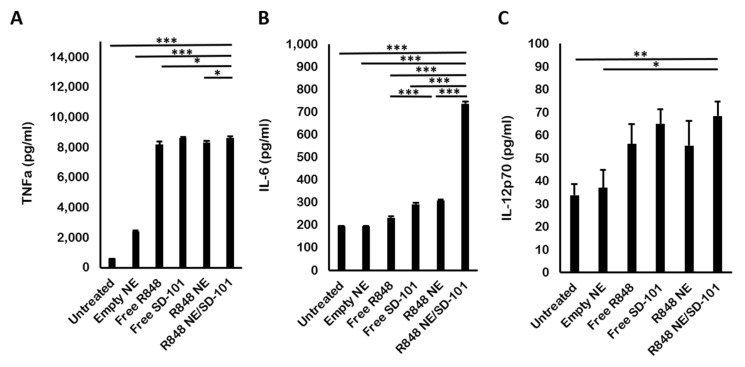
In vitro ELISA for cytokine quantifications. (**A**) TNFa, (**B**) IL-6, and (**C**) IL-12p70 concentrations secreted by RAW 264.7 cells after 12 h treated with complete medium-only, empty NE, free R848, free SD-101, R848 NE, or R848 NE/SD-101 combination. R848 was treated at 50 μM individually, in nanoemulsion, or in combination. SD-101 was given at 300 nM individually or in combination. One-way ANOVA: * *p* < 0.05, ** *p* < 0.01, *** *p* < 0.001.

TNFa is a pro-inflammatory cytokine that is indispensable for early immune response generation. All R848 and SD-101 treatments produced a significant level of TNFa inductions compared to the untreated group. Empty NE also induced a moderate level of TNFa due to the immune stimulation property carried out by squalene (Figure 4A). This result corresponded with the previous research on TNFa induction by TLR7, 8, and 9 activations [42,43]. However, squalene-triggered TNFa production was not significant compared to R848 and R848 NE treatments. In addition, treatments with SD-101 or R848 NE/SD-101 combination showed a slightly higher level of TNFa secretion compared to R848 or R848 NE treatments (Figure 4A), suggesting that TNFa production was favorable in TLR9 activation by SD-101 compared to TLR7/8 activation by R848.

Several reports indicated that both TLR7/8 and TLR9 stimulate IL-6 production, which acts as both a pro-inflammatory and anti-inflammatory cytokine [44,45,46,47]. R848 NE treatment promoted the production of IL-6 compared with R848 treatment, whereas empty NE did not show significant IL-6 production (Figure 4B). It is also noticeable that R848 NE and SD-101, as TLR7/8 and TLR9 agonists, respectively, synergized IL-6 production compared with individual treatments (Figure 4B).

Lastly, IL-12p70 production has been reported to bias Th1 activation resulting in cellular immune responses [48,49]. There was moderate IL-12p70 production in both R848 NE and SD-101 treatment, as monotherapy or in combination (Figure 4C). Squalene-based NE exhibited minor effects on IL-12p70 production stimulated by R848. In addition, the total IL-12p70 levels triggered by SD-101 and R848 NE/SD-101 combination were slightly higher than those triggered by R848 or R848 NE. In contrast to the previous report on the synergistic IL-12p70 production by co-activation of duo TLRs [50,51,52,53], IL-12p70 production was not increased by TLR7/8 and TLR9 co-activation (Figure 4C).

TNFa and IL-12 have been widely known to induce type I T helper cells (Th1 cells) activation, which significantly contributes to antitumor immunity by further activating CTLs and NK cells [54,55]. Although chronic IL-6 signaling could generate a pro-tumor milieu, recent studies also showed that IL-6 can shift the immune responses from T cell suppressive to a responsive state against tumors and also enhance T cell expansion [56]. The elevated expression of pro-inflammatory cytokines (TNFa, IL-6, and IL-12p70) in RAW 264.7 cells treated by R848 NE/SD-101 showed great promise in promoting Th1-biased antitumor activity.

### 3.3. In Vivo Antitumor Efficacy

**Figure 5:**
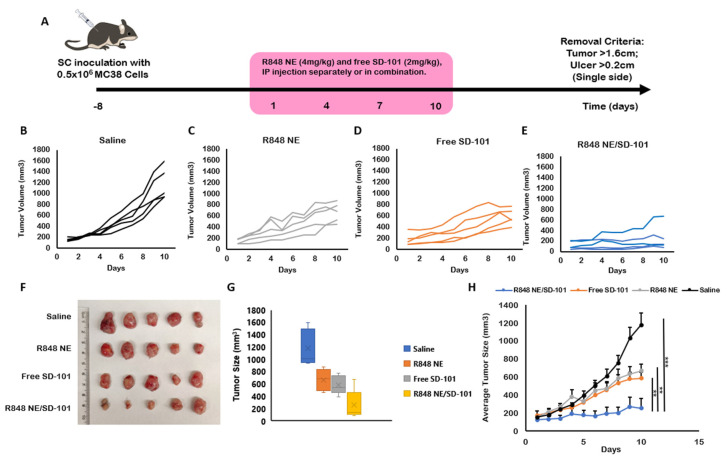
R848 NE and SD-101 treatments of murine colon adenocarcinoma (MC38) syngeneic C57BL/6N mouse model. (**A**) Timeline for MC38 inoculation and treatment regimen. The mice were inoculated with 0.5 million MC38 subcutaneously on the right flank. Treatments began at 8 days after inoculation when tumors became palpable. Treatments were given every 3 days for up to 4 doses. Mice were euthanized after the fourth dose on day 10. (**B**–**E**) Tumor growth over time for individual mice treated with (**B**) saline, (**C**) R848 NE, (**D**) SD-101, or (**E**) R848 NE/SD-101 combination (*n* = 5). (**F**) Images of MC38 tumor tissues were collected at day 10 with measured (**G**) tumor sizes. (**H**) Average tumor growth for each treatment group within 10 days. Data are presented as means ± SEM (*n* = 5). One-way ANOVA: ** *p* < 0.01, *** *p* < 0.001.

It has been shown that TLR7 and TLR9 agonists generate the highest immunogenicity compared with other endosomal TLR agonists by intranasal or oral administration [57]. In addition, intratumoral combination treatment of TLR7/8 and TLR9 agonists was also demonstrated to induce the highest tumor-specific immunity compared with each agent alone [4]. However, intraperitoneal administration is easy, quick, and minimally stressful for both animal studies and patients with metastasis-stage cancer in practice. In the present study, we utilized 4 mg/kg R848 NE and 2 mg/kg SD-101 individually or in combination to show a synergized antitumor efficacy through intraperitoneal administration. The moderate antitumor efficacies by R848 NE and SD-101 individual treatments were observed (Figure 5C,D,F) compared with saline control (Figure 5B), which correspond with the previous studies for R848 and SD-101 [58,59].

Although individual treatments of R848 NE or SD-101 showed significant tumor growth inhibition (TGI) (50.72% ± 16.83% and 65.65% ± 12.88%), R848 NE/SD-101 combination treatment reached approximately 84.62% ± 28.05% total tumor growth inhibition at the end of the study (Table 1) with the median tumor growth inhibition of 98.05% (Table S3), suggesting the synergistic antitumor efficacy carried out by R848 NE/SD-101 combination treatment. No significant differences in body weight indicated mice treated with R848 NE and SD-101 individually or in combination had minor systemic toxicity (Figure S1). Among individual mice treated with R848 NE/SD-101 combination, one mouse was observed with an initial tumor size over 200 mm^3^ which had a slightly higher tumor growth rate compared with other individuals within the same group (Figure 5E). This suggests that additional treatments should be concorded with R848 NE/SD-101 combination strategy to eliminate large solid tumors through intraperitoneal administration.

**Figure 6:**
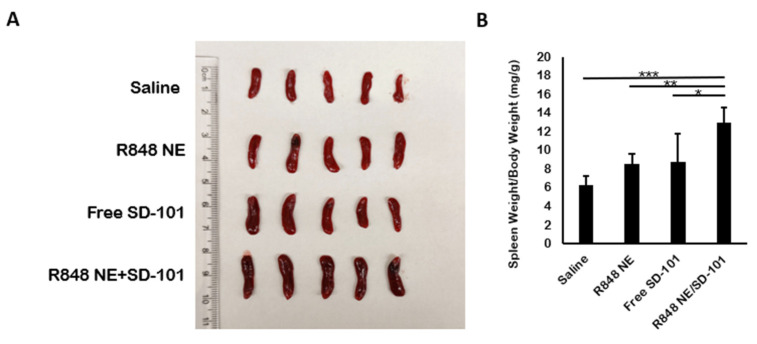
Changes in spleen weights from treated mice. (**A**) Spleen tissues were collected from mice at day 10 with measured (**B**) spleen weights, normalized to individual bodyweight (*n* = 5). One-way ANOVA* *p* < 0.05, ** *p* < 0.01, *** *p* < 0.001.

Splenomegaly indicates T cell activation and NK cell expansion [60]. Besides the significant tumor inhibition in R848 NE/SD-101 combination treatment group, splenomegaly was observed in R848 NE/SD-101 combination treatment group compared with R848 NE or SD-101-only treatment groups as well as the saline control (Figure 6A,B). The observed splenomegaly demonstrated a synergistic antitumor immunity activation carried out by R848 NE/SD-101 combination treatment.

### 3.4. In Vivo Cytokine Production

**Figure 7:**
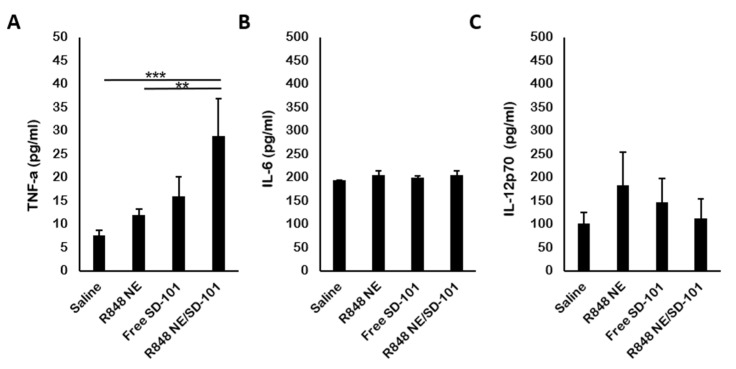
Induction of cytokine by TLR agonists in mice. (**A**) TNFa, (**B**) IL-6, and (**C**) IL-12p70 concentrations were measured in mouse serum by ELISA. Serum samples were isolated from whole blood at day 10 (*n* = 3). One-way ANOVA: ** *p* < 0.01, *** *p* < 0.001.

The presence of TNFa indicates a strong pro-inflammatory cytokine release which further potentiates the tumor cell apoptosis [61]. In mouse sera, a significant increase of TNFa level was observed among mice treated with R848 NE/SD-101 combination (Figure 7A). This, again, demonstrated the synergistic antitumor immunity activation carried out by R848 NE/SD-101 combination treatment along with the remarkable splenomegaly and tumor inhibition. The IL-6 and IL-12p70 productions in vivo differ from those in vitro (Figure 4B,C). No significant change in IL-6 and IL-12p70 level was observed among mice treated with R848 NE and SD-101 individually or in combination compared with saline control (Figure 7C), though TLR7 activation-biased IL-12p70 production was observed in mice treated with R848 NE compared with mice treated with SD-101 or R848 NE/SD-101.

### 3.5. In Vivo Gene Regulation

**Figure 8:**
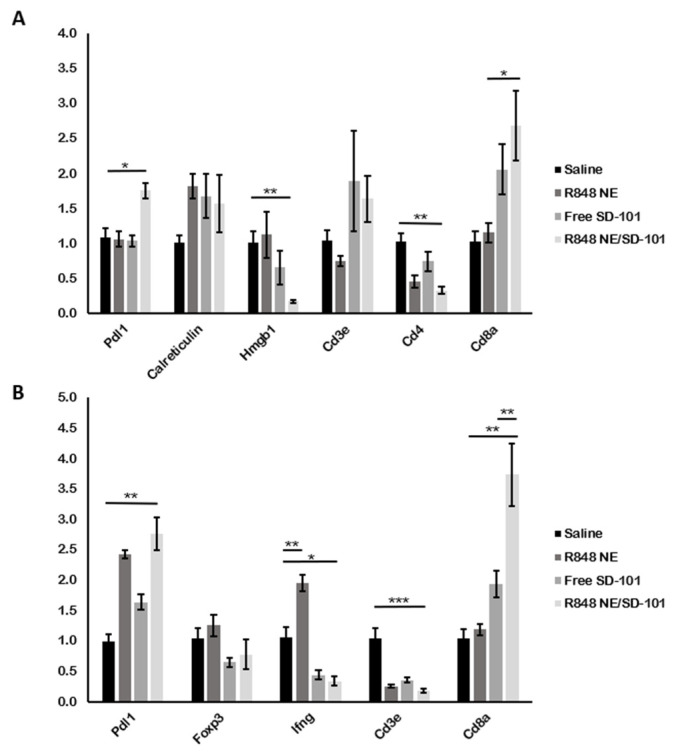
Gene regulation in tumor and spleen tissues from treated mice. (**A**) *Pdl1, Calreticulin, Hmgb1, Cd3e, Cd4,* and *Cd8a* expressions were determined by RT-qPCR in tumor tissues collected from C57BL/6N mice, and (**B**) *Pdl1*, *Foxp3*, *Ifng*, *Cd3e*, and *Cd8a* expressions were determined in spleen tissues collected from the mice within the same study. Data are presented as means ± SEM (*n* = 3). One-way ANOVA: * *p* < 0.05, ** *p* < 0.01, *** *p* < 0.001.

Antitumor immunity carried out by R848 NE/SD-101 combination can be attributed to cellular-mediated cytotoxicities such as CTLs or NK cells activation in concordance with immunogenic cell death (ICD) processes [59,62]. *Cd8a* mRNA was significantly upregulated in both tumor and spleen tissues from mice treated with R848 NE/SD-101 combination along with elevated *Calreticulin* and *Cd3e* (no statistical significance detected) in tumor environment (TME) (Figure 8A), indicating that systemic treatment with R848 NE and SD-101 synergized the antitumor immunity through CTLs and NK cells activation. Different immune regulations were observed in mice treated with R848 NE or SD-101. *Foxp3*, a transcription factor known as the regulatory T cell marker, was downregulated in spleens treated with SD-101 or R848 NE/SD-101 combination, suggesting that fewer regulatory T cells may be generated after TLR9 activation and, therefore, suppress the antitumor immunity by CTLs and NK cells. *Cd3e* and *Cd4* mRNA expressions were significantly lower in tumors from mice treated with R848 NE compared with mice treated with SD-101 (Figure 8A), indicating a lower amount of CD4 T cells infiltrated into TME in mice treated with R848 NE and R848 NE/SD-101 combination. However, the low expression of *Cd3e* and *Cd4* mRNA did not interfere with the high expression of *Cd8a* mRNA in TME (Figure 8A), potentially suggesting that CTLs and NK cells cytotoxicity directed by R848 NE or SD-101 were T helper cell-independent in TME. Considering that Cd8a was significantly upregulated in spleens with downregulated Cd3e (Figure 8B), non-CTLs CD8+ immune cells (NK cells and CD8+ dendritic cells) can also be activated in spleen by R848 NE/SD-101 combination. Treatment with R848 NE also successfully induced *Ifng* expression in mice spleens whereas treatment with SD-101 suppressed *Ifng* expression in mice spleens (Figure 8B). It is possible that SD-101-mediated TLR9 activation increased TNFa production (Figure 7A) which enhanced CTLs and NK cells cytotoxicity in TME. The tumor cell lysis directed by elevated TNFa will further trigger tumor antigen presentation and more rapidly promote immune evasion of CTLs and NK cells in TME [63,64].

*Calreticulin* mRNA upregulation (Figure 8A) was observed in mice tumors treated with R848 NE and SD-101 individually or in combination (no statistical significance detected), where an increase of calreticulin exposure would lead to ICD from tumor antigen phagocytosis by APCs [65]. Secreted HMGB1 from apoptotic tumor cells has been widely known to induce ICD and to enhance tumor cell lysis. However, HMGB1 expression is associated with cell proliferation when cells are not undergoing apoptosis, and it has been shown that knockdown of HMGB1 inhibits tumor proliferation where the release of HMGB1 induces ICD [66,67]. *Hmgb1* mRNA was significantly downregulated in mice treated with SD-101 or R848 NE/SD-101 (Figure 8A), which demonstrated that R848 NE/SD-101 treatment generates a TME that is unfavorable to cell proliferation.

TLR7/8 and TLR9 activation in cancer cells, lymphocytes, and pDCs are also associated with PD-L1 upregulation, which is an immune checkpoint for tumor immune escape [68,69,70,71]. Mice treated with the R848 NE/SD-101 combination exhibited the highest level of *Pdl1* mRNA in both spleen and tumor tissue compared with mice treated with individual agents or saline control (Figure 8A,B). Although people commonly accept the concept of immune escape established by the binding between PD-L1 on cancer cells and PD-1 on T cells [72], more research now indicates that cancer cells can express both PD-1 and PD-L1, and PD-L1 is also found on certain immune cells such as macrophages and dendritic cells [73,74,75]. Considering the role of PD-L1 on antigen-presenting cells that may inhibit T cell function [75], the data suggested that the R848 NE/SD-101 combination treatment through intraperitoneal injection could further exhibit higher antitumor efficacy by incorporating anti-PD-L1 therapeutics to eliminate large tumors or metastatic tumors.

## 4. Conclusions

An R848-loaded squalene-based NE formulation was developed showing better efficiency compared with previously reported liposomal formulations. The R848 NE was shown to be highly stable during long-term storage at 4 °C. An elevated TNFa level in vitro demonstrated strong immune activation by R848 NE and SD-101 individually. Brightfield images and cytokine levels in vitro and in vivo further illustrated a synergistic immune activation when R848 NE and SD-101 were used in combination. Ultimately, R848 NE/SD-101 combination was shown to have potent antitumor efficacy with a TGI of over 80%. Traditional chemotherapy always correlates with high cytotoxicity, which causes dose-limiting damage to normal cells, and novel immune checkpoint blockades are also limited to tumors that have already been infiltrated by T cells [76]. Therefore, a systemic immune response with low side effects is required for effective cancer immunotherapy. Our work indicated that intraperitoneal administration of R848 NE and SD-101 in combination could be a promising approach for CTLs and NK cells-dependent systemic antitumor treatment. Furthermore, the synergized PD-L1 upregulation in vivo by the R848 NE/SD-101 combination suggested a potential therapeutic strategy based on combining R848 NE/SD-101 treatment with anti-PD-L1 therapeutics.

## Figures and Tables

**Table 1:** Tumor growth inhibition (TGI%) at day 10 for R848 NE or SD-101 individually or in combination.

Treatment Group	Mean TGI%	Standard Deviation
R848 NE	50.72	16.83
SD-101	65.65	12.88
R848 NE/SD-101	84.62	28.05

## Data Availability

The authors confirm that the data supporting the findings of this study are available within the article and/or its Supplementary Materials.

## Supplementary Materials

The following are available online at https://www.mdpi.com/article/10.3390/pharmaceutics13122060/s1, Table S1: Cd3e, Cd4, Cd8a, Foxp3, and Ifng primers used in the RT-qPCR for tumor tissues and splenocytes; Table S2: Particle sizes (nm with PDI value) of empty NE and R848 NE at 4 °C for 6-month and at 37 °C for 1-week stability test; Table S3: Tumor growth inhibition at day 10 for individual mouse treated with R848 NE, SD-101, and R848 NE+SD-101; Figure S1: Bodyweight change among mice treated with saline, R848 NE, SD-101, R848 NE+SD-101 during the course.
