# Supplementary material for: A Squalene-Based Nanoemulsion for Therapeutic Delivery of Resiquimod
Source: Pharmaceutics. 2021 Dec 2;13(12):2060. doi: 10.3390/pharmaceutics13122060 (PMC8706843; doi:10.3390/pharmaceutics13122060)
Supplement: Supplementary file 1 [file pharmaceutics-13-02060-s001.zip › pharmaceutics-1468473-supplementary.pdf]

pharmaceutics

Supplementary Materials: A Squalene-Based Nanoemulsion for Therapeutic Delivery of Resiquimod

Zhongkun Zhang, Chun-Tien Kuo, Chi Zhang, Yirui Huang, Zerui Zhou and Robert J. Lee

Table S1. Cd3e, Cd4, Cd8a, Foxp3, and Ifng primers used in the RT-qPCR for tumor tissues and splenocytes.

Target Gene	Forward Sequence (5′ – 3′)	Reverse Sequence (3′ – 5′)	GenBank Number
Cd3e	GCTCCAGGATTTCTCG-GAAGTC	ATGGC-TACTGCTGTCAGGTCCA	NM_007648.5
Cd4	GTTCAG-GACAGCGACTTCTGGA	GAAGGA-GAACTCCGCTGACTCT	NM_013488.3
Cd8a	ACTACCAAGCCAG-TGCTGCGAA	ATCACAGGCGAAGTCCAA TCCG	NM_001081110.2
Foxp3	CCTGGTTGTGA-GAAGGTCTTCG	TGCTCCAGAGACTGCAC-CACTT	BC132333.1
Ifng	CAGCAACAG-CAAGGCGAAAAAGG	TTTCCGCTTCCTGAGGCTG-GAT	BC119063.1

Table S2. Particle Sizes (nm with PDI value) of empty NE and R848 NE at 4°C for 6-month stability test.

	Empty NE	R848 NE
Day 1	145.9 (0.17)	104.1 (0.30)
Day 180	145.8 (0.23)	102.3 (0.34)

Table S3. Tumor growth inhibition on day 10 for individual mice treated with R848 NE, free SD-101, and R848 NE/ SD-101 combination.

Treatment Group	TGI%
R848 NE	30.23
	39.51
	48.89
	64.05
	70.89
Free SD-101	48.12
	56.11
	68.32
	69.25
	81.45
R848 NE/SD-101	36.32
	85.58
	98.05
	99.77
	104.73

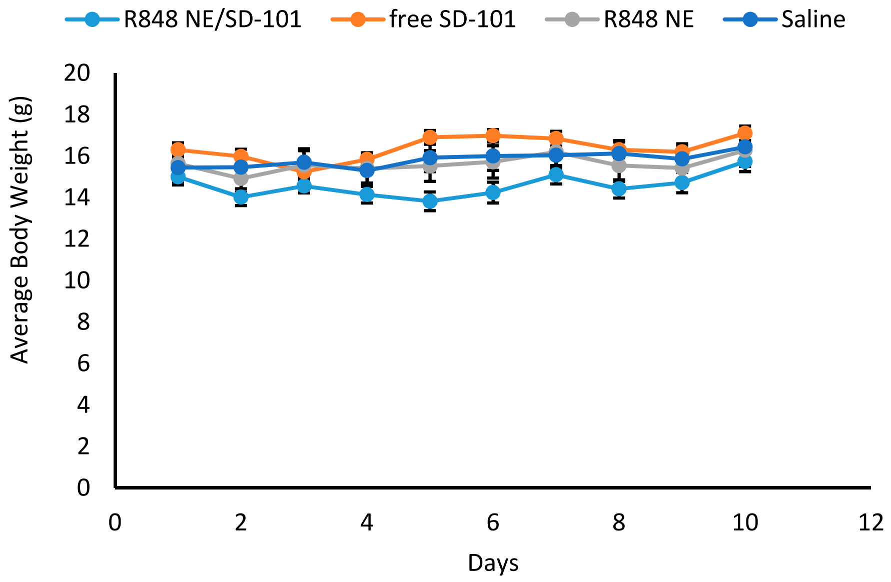

Figure S1. Bodyweight change among mice treated with saline, R848 NE, free SD-101, and R848 NE/SD-101 combination during the course.
